# Evaluating the effectiveness of selected community-level interventions on key maternal, child health, and prevention of mother-to-child transmission of HIV outcomes in three countries (the ACCLAIM Project): a study protocol for a randomized controlled trial

**DOI:** 10.1186/s13063-016-1202-y

**Published:** 2016-02-16

**Authors:** Godfrey B. Woelk, Mary Pat Kieffer, Damilola Walker, Daphne Mpofu, Rhoderick Machekano, the Project ACCLAIM Study Group

**Affiliations:** Elizabeth Glaser Pediatric AIDS Foundation, 1140 Connecticut Avenue NW, Suite 200, Washington, DC 20036 USA; USAID/Bureau for Global Health (BGH)/Office of HIV/AIDS, Washington, DC USA

**Keywords:** MCH, PMTCT, Community-level interventions, Cluster randomized trial, Swaziland, Uganda, Zimbabwe

## Abstract

**Background:**

Efforts to scale up and improve programs for prevention of mother-to-child transmission of HIV (PMTCT) have focused primarily at the health facility level, and limited attention has been paid to defining an effective set of community interventions to improve demand and uptake of services and retention. Many barriers to PMTCT are also barriers to pregnancy, childbirth, and postnatal care faced by mothers regardless of HIV status. Demand for maternal and child health (MCH) and PMTCT services can be limited by critical social, cultural, and structural barriers. Yet, rigorous evaluation has shown limited evidence of effectiveness of multilevel community-wide interventions aimed at improving MCH and HIV outcomes for pregnant women living with HIV. We propose to assess the effect of a package of multilevel community interventions: a social learning and action component, community dialogues, and peer-led discussion groups, on the demand for, uptake of, and retention of HIV positive pregnant/postpartum women in MCH/PMTCT services.

**Methods/design:**

This study will undertake a three-arm randomized trial in Swaziland, Uganda, and Zimbabwe. Districts/regions (*n* = 9) with 45 PMTCT-implementing health facilities and their catchment areas (populations 7,300–27,500) will be randomly allocated to three intervention arms: 1) community leader engagement, 2) community leader engagement with community days, or 3) community leader engagement with community days and male and female community peer groups. The primary study outcome is HIV exposed infants (HEIs) returning to the health facility within 2 months for early infant diagnosis (EID) of HIV. Secondary study outcomes include gestational age of women attending for first antenatal care, male partners tested for HIV, and HEIs receiving nevirapine prophylaxis at birth. Changes in community knowledge, attitudes, practices, and beliefs on MCH/PMTCT will be assessed through household surveys.

**Discussion:**

Implementation of the protocol necessitated changes in the original study design. We purposively selected facilities in the districts/regions though originally the study clusters were to be randomly selected. Lifelong antiretroviral therapy for all HIV positive pregnant and lactating women, Option B_+_, was implemented in the three countries during the study period, with the potential for a differential impact by study arm. Implementation however, was rapidly done across the districts/regions, so that there is unlikely be this potential confounding. We developed a system of monitoring and documentation of potential confounding activities or actions, and these data will be incorporated into analyses at the conclusion of the project. Strengthens of the study are that it tests multilevel interventions, utilizes program as well as study specific and individual data, and it is conducted under “real conditions” leading to more robust findings. Limitations of the protocol include the lack of a true control arm and inadequate control for the potential effect of Option B_+_, such as the intensification of messages as the importance of early ANC and male partner testing.

**Trial registration:**

ClinicalTrials.gov (study ID: NCT01971710) Protocol version 5, 30 July 2013, registered 13 August 2013.

**Electronic supplementary material:**

The online version of this article (doi:10.1186/s13063-016-1202-y) contains supplementary material, which is available to authorized users.

## Background

### Rationale

The global community has set a global target to reduce new pediatric HIV infections by over 90 % and reduce the number of HIV-related maternal deaths by 50 % [1]. Integration of prevention of mother-to-child transmission (PMTCT) services within maternal and child health (MCH) services has become a necessary foundation for transforming service delivery to achieve these goals [2]. HIV programs have successfully improved facility-based service delivery, yet uptake and retention of women in services often lag behind due to structural, social, and behavioral factors [3]. Yet, a preponderance of literature suggests that the social, economic, and cultural systems should be engaged and targeted in order to optimize PMTCT in Africa [4]. Demand for MCH and PMTCT services can be limited by critical social barriers, such as low motivation to seek health services; lack of knowledge or understanding of health issues; lack of information on available services and their importance for healthy families; misconceptions about HIV testing and treatment; fear of disclosure of HIV test results; pernicious HIV/AIDS stigma and the fear of its social effects; harmful gender norms attitudes and behaviors which manifest as poor decision-making ability; limited partner support and participation; and community norms that often preclude open discussion of sexual and reproductive health issues within the family [5–9].

Efforts of PMTCT programs to address these barriers have tended to focus narrowly on HIV and getting the male partner to test at antenatal care rather than more directly as part of pregnancy-related and other MCH care. More importantly, most services related to PMTCT are directed toward women, but fail to address the gender norms that determine women’s participation in these programs [10]. They have also underestimated the role of communities, and of men, in supporting improved health-seeking behaviors by women.

At the household level, male partners have a significant influence over women’s utilization of health services, acceptance of HIV counseling and testing results, ability to adhere to antiretroviral drugs, and infant feeding decisions [11]. However, due to lack of information, men are often ill-equipped to make informed decisions or to take on responsibilities for safe pregnancies and delivery, preventing HIV infection, and helping their families access HIV prevention, testing, care, and treatment services. Women who are able to disclose their HIV positive status to a male partner are more likely to adhere to antiretroviral treatment and to infant feeding recommendations [5]. A limited but growing body of peer-reviewed literature indicates that providing structured social support throughout pregnancy and the breastfeeding period can effectively address shortcomings in medical models of care in high HIV burden settings, thereby helping mothers overcome barriers to care-seeking and adherence [12–14].

Interventions to address these barriers have included male involvement interventions [15, 16], accompaniment models [13, 14], and various community health education and health promotion activities [17]. However, the interventions have generally focused on increasing knowledge, without sufficient attention to addressing prevailing attitudes and norms at the community level, which are essential elements for sustainable behavior change [18, 19]. Moreover, few of these interventions have been systematically tested across varied settings, and there are few instances in the literature testing the joint effects of combined community-level interventions for improved PMTCT outcomes [20]. As with combination HIV prevention, barriers to improving MCH/PMTCT outcomes are often interlinked, so that an intervention approach targeting different levels (community and individual) of the social experience and different social and behavioral constructs (such as awareness, general attitudes and perceived norms) may be more effective than interventions operating on a single element.

A fundamental assumption in the design of this project is that to increase uptake and retention in PMTCT it is necessary to reach all pregnant women, those with and without HIV, as well as their partners, to improve MCH attendance and begin to normalize HIV-related services. This is particularly important with the adoption of lifelong antiretroviral therapy (ART) for all HIV positive women (Option B+) in all three countries and across the region. Option B+ implementation involves same-day initiation of ART, subsequently increasing the need for family and community support to maintain the antiretroviral (ARV) drug adherence and retention in care that is critical to its effectiveness.

In order to address the barriers cited above, we intend to adapt interventions that have demonstrated promise or effectiveness in addressing individual, intrapersonal (peers and family), and community-level barriers to healthcare seeking behaviors. Interventions that have been tested in similar settings (that is, in high HIV-burden, resource-limited settings) were deemed of particular and immediate relevance. Community mobilization through group-based participatory action learning has been demonstrated effective in addressing broader maternal, neonatal, and child health outcomes in a range of resource-limited contexts [21]. A recent meta-analysis of seven women’s participatory groups indicates that these interventions can “…address the exclusion of lower socioeconomic groups from health interventions and help to reach every newborn” [22].

Similarly, the little evidence that exists suggests that dynamic, empowerment-based, solutions-oriented community-wide participation mechanisms bear promise for improving health seeking behaviors [23]. There has been interest in participatory interventions that strengthen the social compact between governments and their citizens — the Global Plan Towards the Elimination of New HIV Infections Among Children (“the Global Plan”) explicitly calls for increased accountability between governments and communities. Interventions aimed at improving the social compact appear promising in the context of primary level service delivery, where communities are closest to the health system [24]. While there are few rigorously evaluated interventions, male involvement is consistently identified as a barrier to retention in the PMTCT cascade [25], with policy, systems, and cultural impediments cited as limiting partner involvement. This trend is reflective of low male uptake of health and HIV services in general. Given this dynamic, novel community-based interventions are a promising vehicle to drive demand and engagement by male partners of pregnant women [26].

This project will systematically implement and evaluate the engagement of community leaders in MCH/PMTCT social action, community health information and promotion events known as community days, and female and male community peer groups in improving the demand for, uptake of, and retention in MCH/PMTCT services.

## Methods/design

### Objectives

The primary objective of this study is to assess the impact of a set of community interventions: the engagement of community leaders, community days, and community peer groups, on the demand for, uptake of, and retention of HIV positive pregnant/postpartum women in MCH/PMTCT services as measured by the proportion of HIV exposed infants returning to the health facility at six to eight weeks of age for the early infant diagnosis (EID) of HIV. The specific objectives are to:

1. Estimate the effect of a set of community interventions that include engagement of community leaders, community days, and community peer groups on the demand for, uptake of, and retention of HIV positive pregnant and postpartum women in MCH/PMTCT services
2. Estimate the effect of community leader engagement on the demand for, uptake of, and retention of HIV positive pregnant and postpartum women in MCH/PMTCT services
3. Assess the added effect of community days on the demand for, uptake of, and retention of HIV positive pregnant and postpartum women in MCH/PMTCT services
4. Evaluate the added effect of the community peer groups on the demand for, uptake of, and retention of HIV positive pregnant and postpartum women in MCH/PMTCT services.

### Hypotheses

**Fig. 1:**
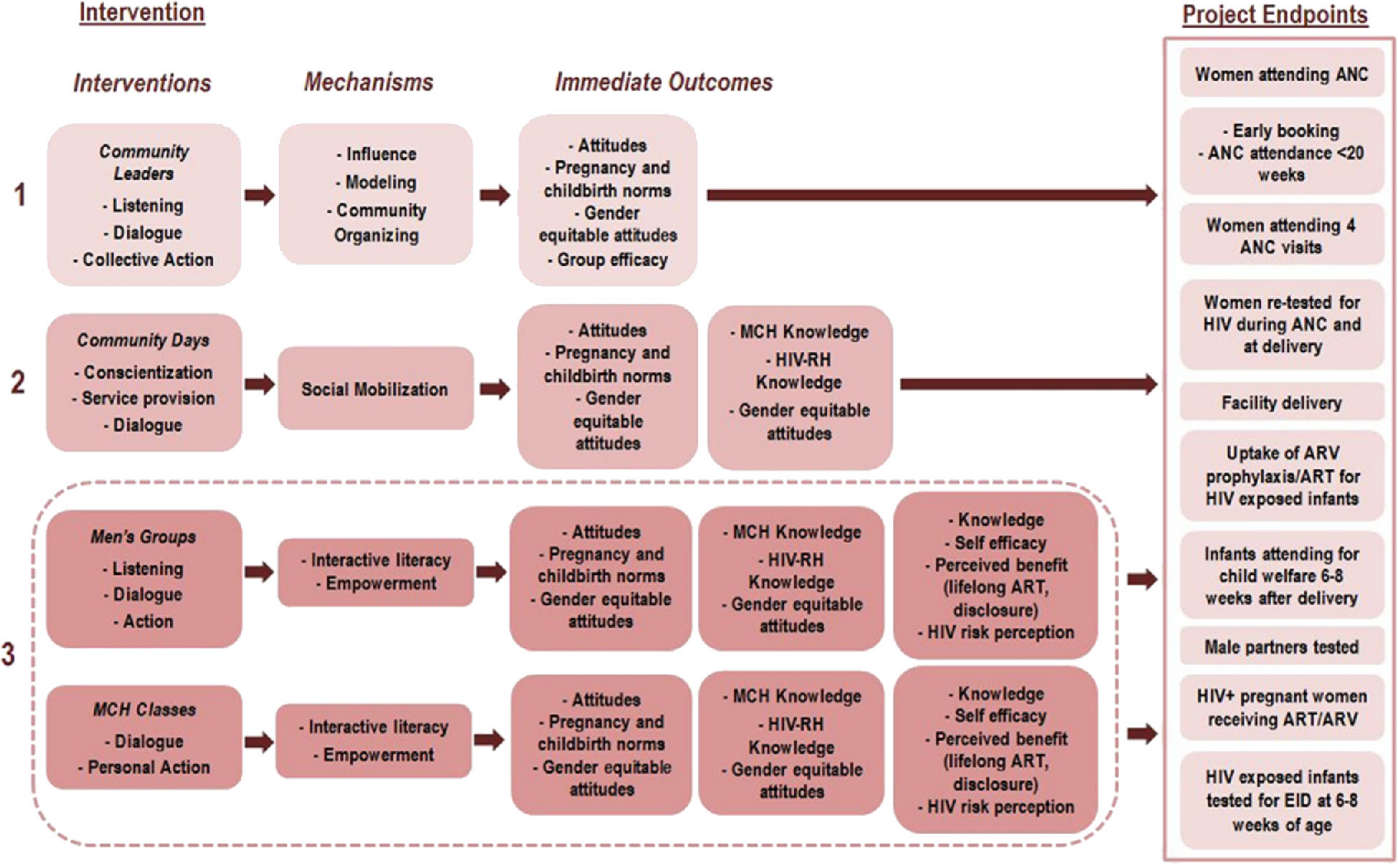
Conceptual framework

We hypothesized that improving individual and community knowledge on MCH/PMTCT and HIV and addressing individual, family, and community socio-cultural and behavioral norms through these selected community-based interventions will increase the number of HIV positive pregnant and postpartum women who are identified, initiated, and retained in PMTCT services. Similarly, we hypothesized that the improvements in knowledge and the focus on norms through these interventions will result in increased utilization of MCH services by pregnant and postpartum women. Guided by a socio-ecological view [27, 28], we further hypothesized that the combined interventions, which target multiple levels of social organization, will be more effective than the individual interventions. Figure 1 presents the conceptual framework.

### Study endpoints and outcomes

The main study endpoints will be derived from service statistics at the health facilities within each cluster (see Table 1).

**Table 1:** Study outcomes

Main outcomes
Retention (primary outcome)
Proportion of HIV exposed infants who present for HIV DNA-PCR testing for early infant diagnosis in the first 6–8 weeks of life.
Secondary outcomes
Demand for services
Proportion of all women presenting for first antenatal care (ANC) visit at less than 20 weeks gestation.
Proportion of all women who complete a minimum of four ANC visits.
Proportion of all women who deliver in a health facility.
Proportion of male partners tested for HIV, of all pregnant women living with HIV
Service uptake
Proportion of HIV exposed infants who receive nevirapine (NVP) prophylaxis at birth.
Proportion of HIV positive pregnant women who receive ART or ARV prophylaxis.

### Design

**Fig. 2:**
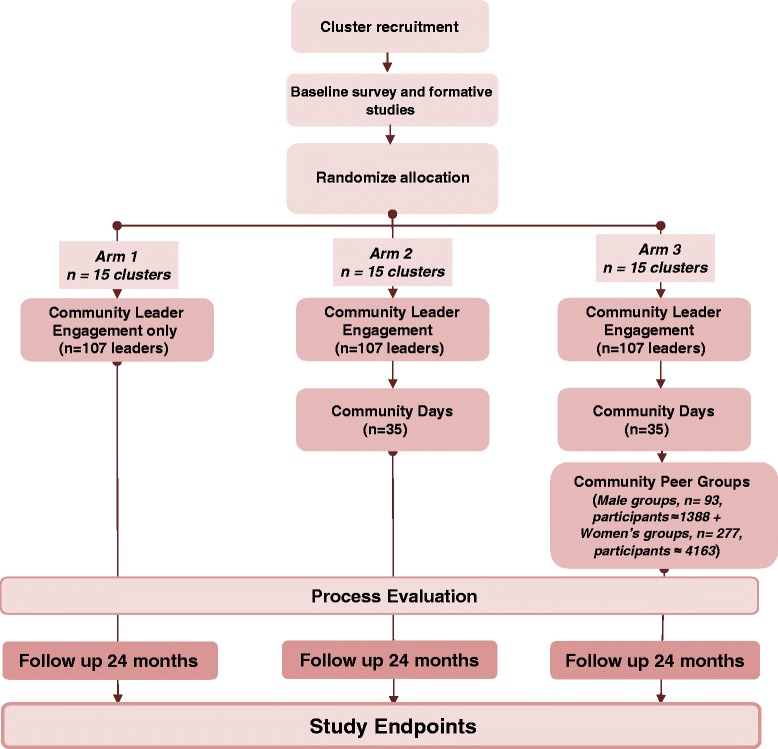
Trial design

This study is designed as a three-arm randomized trial stratified by country: Swaziland Uganda, and Zimbabwe. Within each country, three subunits (regions or districts) were identified, each consisting of 15 clusters (facilities). The regions or districts were then randomly allocated, one to each study arm. In each country, Elizabeth Glaser Pediatric AIDS Foundation (EGPAF)-supported regions with no recent history of intensified community-level PMTCT interventions and low research activity but moderate to high maternal HIV yield were identified as potential intervention areas. The three subunits (for each of the three study arms) were identified corresponding to the administrative structures (that is, regions or districts) existing in that country, also taking into account the ability to implement interventions, with the support of the appropriate health authorities. In each of these districts/regions, five clusters (with a cluster defined as the lowest level of health facility that implements PMTCT, together with its population catchment area [populations 7,300–27,500]) were identified. Criteria for potential selection included recording at least 14 HIV+ pregnant women in the most recent year, the smallest catchment population size, and catchment area completely within the district/region with no overlap, and mix of facility type. Referral facilities and urban facilities were excluded. All clusters meeting these criteria were selected. Where there are more than five per study arm in a country and no logistical concerns to choose one over another, the clusters would then be randomly selected. Each sequentially numbered region/district, with its five clusters, was randomly allocated using random number tables by one of the investigators (GW) in Washington, DC to one of the three arms in the following way: Arm 1 clusters receive an intervention that engages community leaders in leading social action through learning and action cycles; Arm 2 clusters, in addition to the community leader engagement, also implement *community days*, a package that consists of quarterly or semiannual community dialogues and selected health services; Arm 3 clusters, in addition to the community leader engagement and community days interventions, also implement male and female *community peer groups*. Due to the high background levels of community programming in each of these countries (the national PMTCT program in each of these countries includes a robust community programming strategy as part of the minimum essential package of care), the selection of a true control arm was deemed to be both infeasible and unethical. Figure 2 presents the trial design. Additional details are provided in the SPIRIT checklist of recommended trial items in Additional file 1.

At baseline and throughout the intervention, data on the key study outcomes of interest will be collected from the facility MCH/PMTCT quarterly reports. At baseline and at endline, household surveys will be collected to measure changes in key social and behavioral indicators at the community level. Individual knowledge, attitudes, practices, and beliefs will be measured among the community leaders and the peer groups’ members before and after the implementation of the interventions. Finally, individual level health services data will be collected from a selection of the cohort completing the MCH peer groups, as well as from a comparison cohort in the community leader only arm.

#### Formative research

In the design phase, formative work was executed using rapid ethnographic assessment using key informant interviews with formal and informal community leaders, community members, and health workers and observations to: a) map the existing community structures, b) identify influential leadership cadres that could be enlisted as change agents, c) document community perspectives on the gaps that need to be addressed, d) explore the barriers and facilitators to healthcare access, e) identify influences on household decision-making, and f) document community preferences for the intervention settings and schedules. A profile of each community was created using population, health, political, and social indicators.

### Study setting

This study is being conducted in 45 communities in three countries (Uganda, Swaziland, and Zimbabwe). Table 2 presents selected background characteristics of the three countries.

**Table 2:** Selected population parameters for Swaziland, Uganda, and Zimbabwe

Indicator	Swaziland	Uganda	Zimbabwe
Total population, 2012^b^	1,231,000	36,346,000	13,061,239
Total fertility rate (per woman, 2012)^b^	3.41	5.96	3.8
Life expectancy at birth m/f (years, 2012)^b^	55/55	56/58	56/60^g^
Infant mortality rate (per 1,000 live births)^c^	85	54	64
Probability of dying under five (per 1,000 live births, 2012)^b^	80	69	84
Maternal mortality ratio (per 100,000 live births, 2013)^b^	310	360	525
National adult (15–49 years) HIV prevalence, 2012 (%)^b^	26.5 [24.6-28.3]^a^	7.2 [6.4-8.4]^a^	14.7 [13.8-15.6]^a^
HIV prevalence among women 15–49 years (%)	31.1^c^	8.3^d^	17.7 [16.6-18.8]^a, h^
HIV exposed infants tested within 2 months of birth, 2010^e^	54 [47–61]^a^	11 [9–13]	14 [12–16]^a^
ANC coverage - at least four visits (%)^b^	76.6 (2010)	47.6 (2011)	64.8 (2011)^g^
Percent births delivered in health facility^c^	74	57.4	65.1^h^
Gross national income per capita (PPP international $, 2009–2013)^f^	3,080	510	820
GINI index^f^	51.5 (2010)	44.3 (2009)	na
Population living on less than $2 per day (%)^f^	29.3 (2010)	27.4 (2009)	na

^a^95 % confidence intervals

Sources:

^b^WHO country statistics for Swaziland and Uganda (http://www.who.int/countries/swz/en/, http://www.who.int/countries/uga/en/, http://www.who.int/countries/zwe/en/, accessed 16 July 2014), Zimbabwe Population Census Report 2012 (http://196.43.99.13/sites/default/files/img/National_Report.pdf, accessed 19 December 2014)

^c^Demographic and Health Surveys: Swaziland 2006–7, Uganda 2011 (http://www.dhsprogram.com/publications/publication-FR202-DHS-Final-Reports.cfm, accessed 17 July 2014), Zimbabwe Population Census Report 2012 (http://www.dhsprogram.com/publications/publication-FR264-DHS-Final-Reports.cfm, accessed 19 December 2014)

^d^2011 Uganda AIDS Indicator Survey (http://www.dhsprogram.com/Publications/Publications-by-Country.cfm, accessed 17 July 2014)

^e^2010 IATT Fact Sheets (http://www.emtct-iatt.org/countries/, accessed 17 July 2014)

^f^World Bank (http://data.worldbank.org/indicator/SI.POV.GINI, accessed 17 July 2014). [] 95 % confidence intervals, na: Not available

^g^WHO Country Statistics for Zimbabwe (http://www.who.int/countries/, accessed 16 July 2014)

^h^Zimbabwe Demographic and Health Survey 2010–2011 (http://www.dhsprogram.com/publications/publication-FR254-DHS-Final-Reports.cfm, accessed 17 July 2014)

For the purposes of alignment with the existing geopolitical and administrative demarcations, a community cluster is defined as the catchment area surrounding the lowest unit of the healthcare system at which PMTCT services are accessible. The study clusters are almost all rural, but vary in size depending on the zoning system observed by the particular Ministry of Health. The community clusters in Uganda are situated in the southwest region of the country, where the majority of EGPAF Uganda’s operations are based. In Zimbabwe, the study is implemented in Mashonaland East Province. In Swaziland, the study is implemented in Hhohho North and South and Shiselweni Regions.

### The interventions

The community leader engagement intervention will be implemented across all study arms. In Arm 2 facilitated community day events will be implemented in addition to the community leader engagement. In Arm 3 male and female MCH community peer groups will be implemented as well as the community day events and the community leader engagement. Table 3 presents the intervention elements and the contents of the male and female peer groups.

**Table 3:** Intervention themes and elements

Community leader engagement	Community days	Peer groups	
		Men	Women
• Training and capacity building on MCH/PMTCT, gender norms and HIV risk, HIV stigma and discrimination, planning and conducting activities, and community advocacy • Mobilization and dialogue on community solutions to the key health and behavioral gaps as identified from health facility and survey data • Support for the leaders to develop a community action plan (CAP) to organize community-wide social action for MCH/PMTCT.	• Community sensitization and event promotion activities •Large and small group presentations and facilitated discussions • Provision of general health and HIV related services. At minimum this will include: ◦ HIV counseling and testing ◦ HIV prevention information and counseling ◦ Blood pressure screening ◦ Glucose screening ◦Growth monitoring for children (MUAC) ◦Family planning information ◦TB screening ◦Referrals to appropriate health services	• Role of men in family health, including male sexual health • Surgical male circumcision as an HIV prevention mechanism • Chronic and lifestyle conditions • Gender-based violence • STIs, HIV/AIDS, safer sex • Family planning • Disclosure and awareness of HIV status • HIV discordancy among couples • PMTCT • The importance of safe sex during pregnancy • Planning within the family for safe delivery in a health facility • Infant feeding, including the importance of exclusive breastfeeding for the first six months • Infant HIV testing and prophylaxis, and general child health even when the child is well.	• Screening and prophylaxis for syphilis, HIV, tuberculosis (TB), and malaria • Preventive measures during pregnancy (tetanus toxoid immunization, de-worming, folic acid, and malaria prevention) • The importance of HIV testing for women and their male partners, safer sexual practices, the increased vulnerability to HIV during pregnancy and lactation • What PMTCT interventions exist and the support available to women and partners with HIV • Discussing health issues with male partners • Nutrition during pregnancy and prevention of anemia • Danger signs during pregnancy and labor • Preparing for delivery at the facility: birth preparedness and planning for transport • What to expect during labor and delivery, including HIV testing and prophylaxis if necessary • Infant and young child feeding (IYCF): EBF for the first six months • Danger signs during the neonatal period • Infant health: immunization and clinic visits, HIV testing • Postpartum health: family planning and safer sex • Looking after your own health: HIV testing, nutrition, birth spacing, ARV prophylaxis or treatment as applicable
Implemented in all 45 clusters	Implemented in 30 clusters	Implemented in 15 out of 30 clusters	

### Community leader engagement

*Eligibility criteria and recruitment of community leader cadre.* The project preparatory and formative research phases mapped the community leadership structures in the selected clusters in all the countries across traditional, political, civil society, and religious spheres. An intervention component that selects, trains, and equips community leaders to lead social action through a year-long community diagnosis and group participatory process will be implemented across all clusters of the intervention. Consulting deeply and broadly across various social strata of their communities, the community leaders will develop community action plans that explicitly state barriers to desired MCH outcomes, develop personal and community-wide actions and targets to overcome these barriers, and identify the activities and resources (tangible and intangible) necessary to mobilize change. Within the first year of implementation, communities will identify and embark upon at least one social action as documented in the community action plan.

### Community days

At these events community members will be invited to participate in social dialogues, building on a platform of mobile family health services (subsequently referred to as “community days”). Convened on a quarterly basis, this intervention component will be phased in after the social action cycle of the first component (as described above) has been successfully initiated and a draft community action plan exists. Trained community leaders will facilitate community days, drawing upon their extensive social networks and their deep familiarity with local context and norms.

The primary purpose of the community days is to achieve conscientization through open public “discussion space” about MCH issues and especially about the influence of social, cultural, and traditional norms on women’s and child health outcomes. The content of these public dialogues will be documented in a discussion guide based on the four prongs of PMTCT and the related MNCH, reproductive health, and gender issues. Conversational triggers such as the use of group games, music, drama, or film will be utilized (to generate audience interest), followed by a focused introduction (the starter conversation), and will then be summed up in a facilitated plenary group dialogue on the theme being explored. Themes to explore will be linked to the content of community action plans and gaps highlighted in routine facility data.

A package of family health services will be offered to all participants as an incentive for participation. This will include HIV counseling and testing TB (clinical) screening, blood pressure and glucose (urine dipstick) testing, pregnancy testing, information about ANC services and facility delivery, contraceptive counseling, information on medical male circumcision, and child nutrition screening. Other services may be added as appropriate. Attendees with any abnormal results will be referred to the nearest health facility for treatment and/or further investigation.

### Peer group intervention

The community *peer group intervention* consists of peer-facilitated female group MCH classes and male discussion groups.

*Peer facilitated female group MCH classes.* The female group MCH classes will entail four sessions facilitated by a lay experienced woman of similar socio-economic status as the study participants; each session will last 2–3 h. Pregnant women (both HIV negative and HIV positive) will be recruited to the MCH classes (as early as possible in their gestation) from a variety of entry points. Each group will be closed, with membership comprising no more than 8 to 12 women with similar gestational ages (as far as is possible). These sessions will deploy a combination of didactic strategies and participatory mechanisms, such as case studies, role plays, dramas, and songs, to enhance knowledge. Facilitation techniques will support group sharing and peer-to-peer learning, with a focus on encouraging women to share successful strategies for managing their health, household, and community dynamics. The content and facilitation techniques of the female group MCH classes will be manualized for standardization across all study sites. These activities may be conducted at community nodes where women naturally gather (for example, church meetings, women’s groups, markets, water points). Women who miss a session will be followed up at the household level and afforded the opportunity for a catch-up session to ensure their retention in this component.

*Peer facilitated male discussion groups.* The peer facilitated male discussion groups will provide a platform for continuing discussion themes that were initiated at the community day events. Men of reproductive age will be recruited from numerous community entry points (see recruitment section below). Led by a lay facilitator, each male peer discussion group will last about 2–3 h. Over a period of 2–4 months, a total of four sessions will be completed. These activities will take place at community nodes where men gather (such as shops, sporting events, schools, churches, community centers, dip tanks, and under trees). The discussion groups will address issues related to gender and parenting, and will focus on the link between male risk-taking and mother and child health outcomes, with the goal of improving male engagement in health decision-making in their households. The content and facilitation techniques of the male discussion groups will be manualized for standardization across all study sites.

### Intervention timeline

**Fig. 3:**
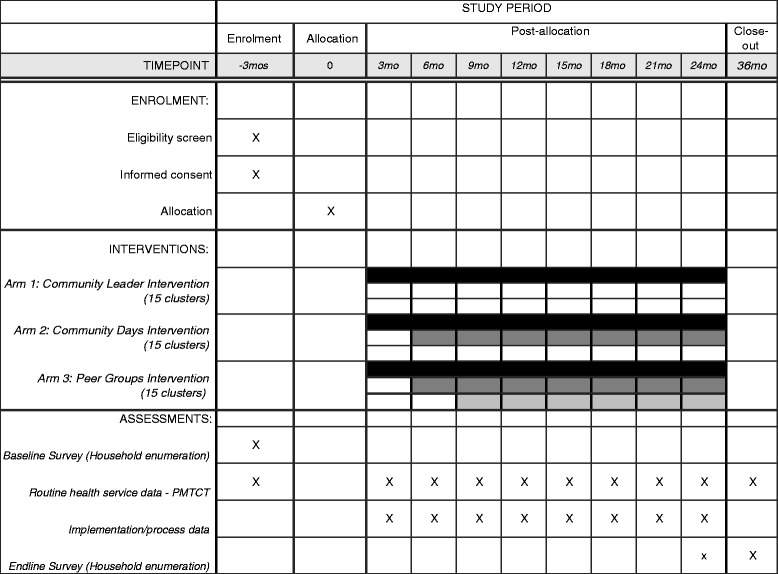
Schedule of enrollment, interventions, and assessments

The community leader engagement intervention will be launched 3 months after the project has begun. Prior to the intervention implementation, the baseline household socio-behavioral survey data collection will have been completed and the routine health services PMTCT data will have been captured. The community day intervention will be launched 3 months after the community leader engagement intervention, and the peer groups 3 months after the implementation of the community day intervention. Figure 3 illustrates the schedule of enrollment, intervention, and assessments.

### Sample size and power

Although this is a three-arm study with the possibility of three between-group comparisons, our sample size estimation is based on comparison between the control group (community leader engagement only) and the group with all three interventions (community leader engagement, community day events, and peer groups). The other two comparisons will be exploratory to estimate the added individual effect of community day events or peer groups.

Based on country program data, we assumed that retention at 6–8 weeks post-delivery (as measured by HIV exposed infants returned for HIV diagnosis at 6–8 weeks) in the control arm will be about 50 %. In a simple randomized control trial, we need 126 HIV positive women per arm in order to detect at least a 20 percentage point increase in retention with 5 % significance level and 90 % power. We estimate that we can enroll at least 14 HIV positive women per health facility. For our cluster-randomized study, we assumed an intra-cluster correlation coefficient for the retention outcome of about 0.01 and an average cluster size of 14 HIV positive women, leading to a design effect of 1.13 and a sample size per arm of 143. With an average of 14 women per facility, this implies 11 facilities per arm. To allow for uncertainty of the intra-cluster correlation coefficient, we increased the number of facilities per arm to 15 (the maximum number of facilities we could afford to enroll in the study). This would allow us to detect the same effect of 20 % with 5 % significance and 90 % power at intra-cluster correlation coefficients as high as 0.05.

#### Community leaders

Across the trial 321 community leaders will be recruited and trained, an average of 7 leaders per cluster. Table 4 presents this information by country. This number of leaders will be selected based on the formative research into community structures and leadership and the feasibility and resources available to train and effectively support them. Efforts will be made to identify formal and informal leaders who have broad reach in terms of being able to mobilize other leaders.

**Table 4:** Expected pregnancies and intervention sample size/recruitment targets

Country	Annual expected pregnancies per cluster (2011)	Estimated HIV+ pregnant women^a^	Women recruitment target (5 clusters)	Number of women peer facilitators	Men recruitment target	Number of men peer facilitators	Number of community leaders
Swaziland	264	82	708	16	236	5	111
Uganda	1,419	118	2,730	61	910	20	120
Zimbabwe	265	47	725	16	242	5	90
Total	1,948	247	4,163	93	1,388	30	321

^a^Based on national prevalence women 15–49 years

#### Community days

Across the countries a total of 70 community days will be held over the 24-month intervention period. The number of community days was estimated based on the desired/possible saturation levels, balanced by the feasibility, logistics, and available resources.

#### Female and male peer groups

The sample size and recruitment targets for women in the MCH classes will be based on reaching 30 % of the projected number of pregnant women by country in order to effect community diffusion of information [29]. Across all the countries 4,163 pregnant and postpartum women will be recruited into the MCH classes, with Uganda contributing more than half of that number due to its larger cluster population sizes and higher fertility rate. The number of men in the male discussion groups was based on recruiting at least 30 % of the number of women in the MCH classes, for a total of 1,388 men. We based this number on the feasibility and practicality of recruiting these many men and on the resources available. As for the peer facilitators of these groups, we estimated that a peer facilitator could manage a group of up to 15 participants at a time and be able to conduct three groups during a year, so that we will recruit 93 peer facilitators for the women’s groups and 30 for the men’s group overall.

### Recruitment

#### Study participants

Information about the study interventions will be disseminated broadly through various community outlets and channels (with the exclusion of mass media) in order to optimize community participation in the study interventions. For the peer-based interventions, study activities will be disseminated through these channels, with the community day events serving as a seminal recruitment opportunity. In addition, more specific entry points will be targeted, including PMTCT clinics, village health workers (VHWs), traditional birth attendants (TBAs), and community leaders for recruiting women to the *MCH classes*, and ANC partner invitation letters, health facility referrals, peer nominations (snowballing), and public notices for recruiting men to the *male discussion groups*.

Women will have to meet the following inclusion/exclusion criteria to participate in the project: 18 years and above; pregnant; resident in the community defined as having lived there for at least 3 months prior to enrollment; intention to remain in the area until 8 weeks postpartum; ability to attend the MCH sessions; ability to give informed consent. Exclusion criteria are: non-resident; unwilling or unable to attend the group sessions. For men inclusion/exclusion criteria for joining the discussion groups include: 18–60 years of age; resident in the community for at least 6 months prior to enrollment; intention to remain in the area for the duration of the intervention (approximately 2–4 months); able to attend the group sessions; ability to give informed consent.

Participants who are actively or recently enrolled in an intervention to assess study outcomes that are similar to those specified in this protocol will be excluded. No monetary compensation will be provided for participation in any of the study interventions.

#### Intervention facilitators

Three types of lay cadres will be selected, trained, and equipped to deliver the selected interventions to the target audiences. Nominations will be selected from community leaders, community health workers, health staff, and relevant community-based organizations (CBOs)/non-governmental organizations (NGOs). For the social action component, *community leaders* will be selected according to the following criteria: current key leadership role in community institutions of any type; interest in and commitment to promoting the visibility of maternal and child health issues including safe motherhood and the elimination of pediatric HIV; motivation to work across organizations to support community action for maternal and child health issues; long-term residency (minimum of 2 years and preferably at least 5) in local area and current residency in the area; appropriate educational levels; native fluency in the main local language; ability to read and write. Representatives of the PLHIV constituency youth and/or women leaders will be given priority. Criteria for selection as a male peer facilitator include at least 4 years of secondary education, willingness to participate in the project, availability for the project period, good communication skills, and equitable gender attitudes. Women who are living with HIV and have recently completed enrollment in PMTCT services will be prioritized if possible. Peer facilitators will be invited to a 5-day training course (with a 2–3 day refresher training after the first round), which will include research ethics and informed consent procedures as well as the importance of confidentiality. The peer facilitators will also be required to sign confidentiality agreements. Facilitators will be modestly remunerated.

The community leaders, peer group participants, and peer group facilitators can choose to exit the project at any time, without prejudice to services or benefits.

### Data collection

Data on study outcomes (outlined in Table 2) will be collected from numerous sources, including routine health service records, a household socio-behavioral survey, and process monitoring and assessments.

#### Routine health service records

Data on the primary and secondary study outcomes of interest will be abstracted quarterly from health facility reports upon completion of a data verification exercise. Trained data collectors will also collect individualized data from the medical records and/or registers for women who are recruited into the female MCH classes. As the main endpoints of this study are dependent on routinely collected health facility (services) data, it is critical that these data are valid and reliable.

To ensure the data validity, we will implement a process focused on ensuring the quality of data captured in the health facility registers and reports. Where significant differences are observed (>10 % deviation on any variable), training will be provided to the staff at that facility. Facilities which exhibit high error rates will be monitored monthly until improvement. We will undertake this activity immediately prior to the implementation of the intervention and just prior to finalization of the intervention activities. To examine trends over time, project staff will assess the data for the 3-month period before intervention implementation, and similarly for the midpoint and endline assessments.

These datasets will be entered at the site level into the GLASER database, an EGPAF global database managed centrally (in Washington, DC). This data will undergo several levels of validation at country and global levels, after which it will be exported into a statistical package such as STATA, SAS, or SPSS for further analysis.

#### Household socio-behavioral survey

*Men and women aged 18*–*60 years* were surveyed enumerated to establish baselines variables for key mediators and moderators of the health outcomes of interest. Households were randomly selected based on sampling frames drawn from census lists and augmented by household lists or maps held by community leaders and/or the relevant administrative offices. The surveys were enumerated by trained research assistants with appropriate supervision, and the data were captured directly into an EPIINFO v7.1 database on laptop computers in the field. The survey was translated into the local languages (Runyankole, SiSwati, and Shona) and pre-tested. Items from the following validated scales and indicator compendiums were included:

*Gender Equitable Men* (*GEM*) *Scale* [30]. A 24-item scale with two subscales (gender equitable and inequitable attitudes) that measures attitudes toward gender norms in intimate relationships or differing social expectations for men and women. We retained four domains (violence, sexual relationships, domestic chores and daily life, and reproductive health and disease prevention). The GEM Scale has been used and adapted successfully in over seven different countries in Asia, Africa, and Latin America, and is considered to be cross-culturally relevant. In psychometric assessment (factor analysis), Cronbach’s alpha was 0.81. Tests of internal consistency yielded an alpha of 0.88 in an Ethiopian adaptation.

*Tanzania Stigma Indicator and Community Baseline* (*Individual Questionnaire*). This model questionnaire [31] was developed for assessing community stigma levels, drawing upon existing indicators from USAID’s Expanded Response Guide to Core Indicators for Monitoring and Reporting on HIV/AIDS Programs (the “Blue Book”) and indicators developed by USAID’s Stigma and Discrimination Indicators Working Group. The sections of this questionnaire include *Knowledge of HIV*, *Shame and Blame*, *Enacted Stigma*, *Disclosure*, and *Knowledge of Policies and Laws*, and it was validated in Tanzania. Together, they assess the four domains of stigma: fear of casual transmission, values (shame, blame, and judgement), enacted stigma, and disclosure. We adapted items from the *Knowledge of HIV, Shame and Blame,* and *Enacted Stigma* sections. In a validation study in Nicaragua [32], an abbreviated tool with a subset of the stigma items recommended in the Tanzania model questionnaire underwent psychometric testing. Evidence of internal consistency was found (Cronbach’s alpha of 0.81 for the HIV stigma scale and 0.91 for the HIV discrimination scale).

*World Bank Social Capital Assessment Tool* (*A*-*SCAT*) [33]. A modification of an extensive scale comprising three modules (the community profile household survey and organizational profile), the items of the A-SCAT have been tested for use in India and Panama, and elements of the tool tested further in Peru and Vietnam. The A-SCAT is intended to assess, at the individual and community levels, the multidimensional nature of social capital, including structural and cognitive components. We incorporated items from the household survey module of the A-SCAT, specifically items on organizational density and characteristics, and networks. In psychometric validation of an abbreviated version (the SASCAT - short version of the Adapted Social Capital Assessment Tool) completed in Peru and Vietnam [34], the strongest factor was displayed by the questions on *group membership*/*social support*, with an alpha of >0.80 in Peru and Vietnam. The SASCAT clearly distinguished the different concepts comprising social capital, though refinements were suggested to enable discrimination between support from individuals and group membership.

*Rapid CATCH+ 2009 Maternal and Neonatal Care Module* [35]. We selected items from Child Survival Technical Support/MCHIP’s Knowledge Practices and Coverage Survey (KPC), a rapid small population based survey that was originally developed by Johns Hopkins University and has been used by USAID Child Survival and Health Grants Program (CSHGP) grantees since 1991, with periodic revisions. The tool is traditionally used with mothers of children 0 to 23 months (limiting recall bias). We selected items to assess knowledge and practices related to appropriate household care and health-seeking behaviors for the mother-baby pair during pregnancy, labor, and the postpartum/neonatal period.

#### Process monitoring and assessments

We intend to assess the extent to which interventions are implemented as planned to enable appropriate inferences to be drawn from the effectiveness evaluation, as well as to document other activities/projects that may impact the study implementation and findings. Various types of deviations from design are well documented in the literature. Of interest to us are the adequacy of required inputs, sufficiency of enrollment and program coverage (”dose”), retention/completion rates, and adequacy of change on immediate and intermediate outcomes [36]. We will develop a process to monitor the implementation of the study, in particular the extent to which the interventions are implemented as planned. At least once during the study, EGPAF staff unaffiliated with the project will audit the trial using a structured instrument, with these forms being collated by the program coordinator for review by the project management team. Various intervention facilitators will be trained and equipped to document intervention delivery through attendance records and routine reports. Intervention facilitators will document the intervention elements (such as number of community days, number of communities with a completed community action plan, referrals initiated, number of men or women completing the peer group cycle) through program records and routine reports, which will be verified during supervisory and site visits. Intervention facilitators will also monitor for any potential adverse events (for example, spouse’s threatening harm to their partner for attending peer group meetings), through sensitization of peer facilitators and supervision activities, with documentation on study approved forms. The effectiveness of facilitator trainings and peer group activities will be assessed through structured pre-/post- questionnaires administered by research assistants. Client exit interviews will be embedded into community days to assess the changes to MCH and HIV related immediate outcomes (knowledge, attitudes, and beliefs), and we will assess the completion of referrals initiated during the community days by independent verification at health facility level.. Finally, we will systematically inventory new, related activities by other organizations and research projects within the project clusters, and update this information on a quarterly basis.

### Data management

Summary data will be extracted from the routine health facility reports after they have been submitted to the Ministry of Health. Each quarter, data for study endpoints which are not routinely reported at the district/national level will be extracted by project staff directly from the health facility registers in de-identified form. All study data will be entered into custom modules in GLASER, a database managed in Washington, DC.

An Epi-Info v7.1 database will be developed for the intervention monitoring and process evaluation data. These data consist of data elements monitoring the implementation of the interventions, enrollment and program coverage, retention and completion rates, and assessment of intermediate outcomes. The forms and tools capturing enrollment, program coverage, and retention/completion rates will be collected by project staff and peer facilitator supervisors for entry into the Epi-Info v7.1 database. The paper-based KAPB questionnaires from the community leaders and peer group participants (intermediate outcome assessment) will be transported by project staff to the project offices, where they will be entered into the Epi-Info v7.1 database. The adequacy of project inputs and unanticipated events will be largely captured in monthly operational reports.

In each country, monitoring and evaluation officers will maintain the databases. On receipt of the data, they will perform data quality checks (data completeness, consistency) according to an algorithm already developed for program data and one to be developed for the process data. They will also ensure the security and integrity of the databases through password protection of the data, limited access to the site where the data will be housed, and regular backup of the data. The officers will run quarterly reports of the health facility and process data for the country project team to monitor implementation of the project.

Encrypted data from all the countries will be transferred to the databases in the EGPAF global office in Washington, DC. The project statistician will facilitate data merge, and access to the data will be limited. The project management team will review the data regularly and at least monthly to monitor project progress and implementation. The project management team consists of the principal investigator, project director, deputy project director/program coordinator, community advisor, biostatistician, and financial analyst. The team meets weekly to monitor progress on the project.

### Human subjects protection, ethical considerations, and dissemination

The study protocols were submitted for ethical review and approved by the Medical Research Council of Zimbabwe (MRCZ), MRCZ/A/1707, 19 Dec. 2012, and MRCZ/A/1776, 7 Oct. 2013, the Swaziland Scientific and Ethics Committee (SEC), MH/599C, 9 Apr. 2013 and 23 Oct. 2013, the Uganda National Council on Science and Technology (UNCST) National HIV/AIDS Research Committee (NARC), SS3057, 15 Feb. 2013 and ARC146, 25 Oct. 2014, and the Institutional Review Board (IRB). Ethical approval has been granted by all participating review boards in the three countries. Amendments to the protocol have been sought from and approved by the IRBs, and these changes communicated to the relevant authorities. All communities were apprised of the goals and aims of this study, and assent was secured verbally by the community leaders as well as the Ministries of Health and other relevant government authorities. In addition, individual participants were thoroughly briefed on the study objectives and activities; consenting was conducted in the native language by trained research assistants, and informed consent secured in writing prior to participation in any activities requiring collection of individually identifiable data. (A draft informed consent form for women attending the MCH classes is provided in Additional file 2.) All members of the research teams were trained and certified in human protections research.

Trial results will be disseminated through public meetings in the community sites, publications in the peer reviewed literature, conference abstracts, and presentations. Reports on the study will follow the CONSORT statement [37] as well as its extension for cluster trials [38]. Access to individualized study data will be restricted to members of the ACCLAIM study group and to the regulatory authorities. Routine study service statistics will be available for further analysis to staff members of the Elizabeth Glaser Pediatric AIDS Foundation who routinely support clinical service programs in the participating countries.

### Data analysis

The overall objective of this study is to estimate the effect of a package of community interventions on retention in care, demand for, and uptake of PMTCT services among HIV positive women. The primary specific aim of the study is to estimate the effect of a package of interventions that include engagement of community leaders, community days, and community peer groups on retention in care assessed by the proportion of infants born to HIV positive women tested for HIV within 6–8 weeks post-delivery (EID). The secondary aim of this evaluation study is to estimate the added effect of each intervention separately.

#### Primary specific aim

This study uses health facility quarterly aggregate data collected routinely through the usual monitoring and evaluation systems. The effect of community days and community peer package on retention will be estimated by comparing the average proportion of infants tested for EID between study Arm 1 against study Arm 3 during the quarter immediately following the ending of the interventions. Since the implementation of the interventions started at different times for the different countries, the intervention will be expressed as the amount of time during which a health facility was exposed to an intervention package. The analysis will also take into consideration that health facilities are clustered within districts and stratification of the intervention arms by country. We propose the following mixed effects model to estimate intervention effects:

$$ logit\left({\pi}_{ijk}\right)={\beta}_0+{\beta}_1{T}_{ijk}+{\beta}_2{C}_k+{\beta}_3{\boldsymbol{X}}_{ijk}+{\eta}_{jk} $$

where *π*_*ijk*_ is the proportion of HIV exposed infants at facility *i* in district *j* in country *k* tested for HIV at 6–8 weeks, *T*_*ijk*_ is the time of exposure to the full intervention package of community leaders, community days, and community peer groups for facility *i* in district *j* in country *k*, *C*_*k*_ is the country indicator variable modeled as a fixed effect, ***X***_*ijk*_ is a vector of health facility level and district level covariates including proportion of infants tested for HIV within 6–8 weeks before the implementation of the intervention, and *η*_*jk*_ is the random effect for district *j* in country *k*. The observed proportions *P*_*ijk*_ are assumed to have a binomial distribution. The regression coefficient *β*_1_ estimates the effect of exposure to the package of intervention.

#### Secondary aim 1

To estimate the effect of community leaders on early infant diagnosis, we will compare the proportion of exposed infants tested for HIV within 6–8 weeks before the introduction of community leaders to the proportion of exposed infants tested for HIV within 6–8 weeks at the end of intervention implementation using quarterly data. For each facility, the difference between the before and after implementation community leader intervention and its associated standard error will be computed. The facility specific differences will be pooled together using an inverse variance weighted mean accounting for the design of the study. This analysis will be restricted to 15 facilities assigned to the community leader only arm.

#### Secondary aim 2

The added effect of community days will be estimated by comparing facilities in study Arm 1 (community leaders only) to facilities in study Arm 2 (community leaders plus community days). A model similar to the one described above for the primary aim, where *T*_*ijk*_ is the time of exposure to the package of community leaders and community days for facility *i* in district *j* in country *k*, will be estimated.

#### Secondary aim 3

The added effect of community peer groups will be estimated by comparing facilities in study Arm 2 (community leader and community days) to facilities in study Arm 3 (community leaders, community days, and community peer groups). *T*_*ijk*_ will be the time of exposure to the full package of community leaders, community days, and community peer groups in the model described above.

#### Secondary outcomes

All analyses described above will be repeated for secondary outcomes, which are also measured at the facility level as proportions. We are not particularly concerned with testing for significance on these secondary outcomes but on the estimation of the effect of the interventions and associated confidence intervals. We therefore do not propose any *p*-value adjustment to account for multiple testing.

As indicated earlier, we propose to assess changes in key social and behavioral indicators at community level through the baseline and endline household KAPB surveys. We will analyze changes through evaluating differences in responses to these indicators between the pre- and post-surveys by country. Statistical differences in proportions and means will be assessed using chi-square tests or *z*-tests as appropriate, with a statistical cut-off of *p* < 0.05.

## Discussion

The protocol is currently being implemented, and this has necessitated changes in the original study design to accommodate practicalities on the ground. In the original protocol we had proposed that the clusters would be randomly selected. However, with the selection criteria of the lowest level facility undertaking PMTCT and recording at least 14 HIV positive pregnant women annually, as well as avoidance of overlapping catchment areas, too few facilities were left to randomize. Thus, it was more practical to purposefully select the facilities where, for example, there were two facilities that met the original criteria, based on clearly defined catchment areas.

As the study was implemented, first Uganda, then Zimbabwe, and finally Swaziland implemented Option B+, lifelong antiretroviral therapy for all HIV positive pregnant and lactating women. In all three countries, implementation of this program occurred rapidly so that all the ACCLAIM study areas were carrying out Option B+ within a period of a few weeks. Thus, there was no difference across the study arms with limited/no potential for it to differentially impact the study outcomes. In addition, monthly study teams monitor and document activities which may impact the study, such as any new research projects which may have similar goals, relevant changes in health services, and national health campaigns. These reports are regularly reviewed by the study management team, which in any case has weekly meetings. Data on potentially confounding activities and events will be incorporated into analyses at the conclusion of the project. As part of the study preparations and permissions, regional/provincial and district authorities have been informed of the proposed study and their support for the success of the study solicited.

A strength of this study is that it tests multilevel interventions in overcoming critical social barriers to improving demand for, uptake of, and retention in MCH/PMTCT services. The protocol also utilizes program data as well as study-specific and individual level data. This trial is also conducted under “real world” conditions, using program support and infrastructure; thus, the findings are more likely to be robust.

A limitation of the protocol is that there is no true control arm given the limitation of resources and the potential ethical considerations. In all three countries, community consultation and support are necessary to carry out any community-based research. Thus, if a fourth control arm had been developed, there would have been significant overlap with the community engagement arm. Though not optimal, we intend to undertake a before/after analysis in the community leader engagement only arm to assess for the potential effect of this intervention to mitigate the limitation of the lack of a true control arm.

Another limitation is that the protocol does not adequately control for the potential effect of the implementation of Option B+. Option B+ would intensify messages such as early ANC and the importance of male partner testing, and this may potentially confound the findings, particularly in the community engagement intervention arm. To potentially control for this, we will analyze program and surveillance data for selected variables in EGPAF-supported sites in districts/regions that are not in the ACCLAIM project. For example, Zimbabwe has an electronic patient level data system in selected sites, which could be used for this purpose.

## Trial status

Enrollment is anticipated to end by December 2015.

## Additional files

Additional file 1:
**SPIRIT 2013 Checklist: recommended items to address in a clinical trial protocol and related documents.** (DOC 122 kb) Additional file 2:
**Consent Form, Female MCH Classes; Project ACCLAIM: Advancing Community Level Action for Improving MCH/PMTCT.** (DOCX 23 kb)

## Abbreviations

ART: Antiretroviral therapy
EID: early infant diagnosis
EMTCT: elimination of mother-to-child transmission (of HIV/AIDS)
L&D: labor and delivery
MCH: maternal and child health
MRCZ: Medical Research Council of Zimbabwe
NARC: National HIV/AIDS Research Committee (Uganda)
PCR: polymerase chain reaction
PMTCT: prevention of mother-to-child transmission (of HIV/AIDS)
SEC: Scientific and Ethics Committee (Swaziland)
UNAIDS: UN Agency for International Development
WHO: World Health Organization
