# Supplementary material for: Evaluating the effectiveness of selected community-level interventions on key maternal, child health, and prevention of mother-to-child transmission of HIV outcomes in three countries (the ACCLAIM Project): a study protocol for a randomized controlled trial
Source: Trials. 2016 Feb 16;17:88. doi: 10.1186/s13063-016-1202-y (PMC4754877; doi:10.1186/s13063-016-1202-y)
Supplement: Additional file 2: — Consent Form, Female MCH Classes; Project ACCLAIM: Advancing Community Level Action for Improving MCH/PMTCT. (DOCX 23 kb) [file 13063_2016_1202_MOESM2_ESM.docx]

**Consent Form**

**Female MCH Classes**

*Project ACCLAIM*

*Advancing Community Level Action for Improving MCH/PMTCT*

Principal Investigator: ______________

Phone number: _____________

**INTRODUCTION:**

Hello, my name is____ I am from the Elizabeth Glaser Pediatric AIDS Foundation. Our organization works on mother and child health and preventing the transmission of HIV from mothers to their babies. I would like to talk to you about a study we are doing on how to improve mother and child health services including the prevention of mother to child transmission of HIV. We are doing this study with the support of the Ministry of Health.

**PURPOSE**

You are being asked to participate in a research study. The purpose of the study is to increase community use of Maternal Child Health (MCH) and Prevention of Mother-to-Child transmission of HIV (PMTCT) services. You were selected as a possible participant in this study because you are a woman from the community we would like to learn more about.

This study will take place in three countries; Uganda, Swaziland and Zimbabwe. Altogether we expect to have about 4163 women in this part of the study, with about xx of the women from this country.

**PROCEDURES AND DURATION**

If you decide to participate, information about you and your pregnancy will be collected and you will be asked to attend group meetings held monthly. There will be a total of 4 group meetings, each lasting about 3 hours.

A peer facilitator will provide you with more information on pregnancy and childbirth, and care of the young child, and as a group you will be able to freely discuss topics on the importance of early booking, nutrition during pregnancy, benefits of health facility delivery, prevention of mother-to-child transmission (PMTCT) of HIV, ANC and post-natal care.

At the end of the all the group meetings, we may ask your opinions about the group meetings and the group process, and this interview may last for an additional 15 minutes.

Your attendance at these meetings will be very important for you to learn about how to take care of yourself and your baby and for our study. You may be visited at your home by the peer facilitator if you miss some meetings so they can find out if there are any problems. Also, study staff will look at health records in the antenatal, delivery, and postnatal clinics and collect information from the clinic that may include your health information.

Also, if your spouse or partner attends the maternal and child health classes, we may try and relate your information and records with his information.

We will also ask about your views and knowledge on sexual and reproductive health, mother and child health, HIV and sexually transmitted diseases. You will be asked also about norms relating to the roles of men and women. This interview will take approximately 30 minutes and will take place at the beginning of the first group meeting, and again at the final group session.

**RISKS AND DISCOMFORTS**

We do not expect any risks or discomforts to the study participants. You may however feel uncomfortable with some of the topics we talk about. You do not have to answer any questions that make you uncomfortable. There is a risk that your information may be disclosed to unauthorized people. We will every effort to ensure that this does not happen and we will not include your name on the reports that we will write, so that no one will know about your information specifically.

**BENEFITS AND/OR COMPENSATION**

There may be benefits for you and your child for participating in this study. In your group meetings you will receive advice and information. The information will be about having a healthy pregnancy, labor and delivery and keeping your child healthy. You will be able to ask questions and discuss anything you have heard in the community.

**CONFIDENTIALITY**

Confidentiality will be assured to the best of our ability. Your name will not be in the writing on study papers, or used in any report. This signed consent form with your signature will be kept separate from other study papers in a safe, locked place. Only ethical committee members, study members, and people from the group that paid for the study may look at study records. Research staff at the Elizabeth Glaser Pediatric AIDS Foundation may have access to these data. Staff members who have access to your personal information will be trained in how to protect your privacy and keep your information confidential.

**ADDITIONAL COSTS**

There are no additional costs to participate in the study. The study will be done in your community so that there are no transport costs.

**IN THE EVENT OF INJURY**

We do not anticipate any injury, harm or illness from participation in the group discussions or the interview. In the event of injury resulting from your participation in this study, treatment can be obtained at your local clinic. You should understand that the costs of such treatment will be your responsibility. Financial compensation is not available.

**VOLUNTARY PARTICIPATION**

Participation into the study is voluntary, but we hope you will decide to take part. If you decide not to participate in this study, your decision will not affect your future relations with the clinic**.** If you decide to participate, you are free to withdraw your consent and to discontinue participation at any time without penalty. You are free to refuse to answer any questions that make you uncomfortable.

**OFFER TO ANSWER QUESTIONS**

**Tick one box:** ⁫ Participant is literate ⁫ Participant is illiterate

Before you sign this form, please ask any questions on any aspect of this study that is unclear to you. If you need time to think this over, please let me know.

If you have any questions about this study, you may ask the Principal Investigator, _______ on telephone _______

If you have any questions concerning this study or consent form beyond those answered by the investigator, including questions about the research, your rights as a research subject or research-related injuries; or if you feel that you have been treated unfairly and would like to talk to someone other than a member of the research team, please feel free to contact the _________ (IRB/Ethics review committee) on telephone ______ or _____.

**AUTHORIZATION**

You are making a decision whether or not to participate in this study. Your signature indicates that you have read and understood the information provided above, have had all your questions answered, and have decided to participate.

[The date you sign this document to enroll in this study, that is, today’s date, MUST fall between the dates indicated on the approval stamp affixed to each page. These dates indicate that this form is valid when you enroll in the study but do not reflect how long you may participate in the study*.* Each page of this Informed Consent Form is stamped to indicate the form’s validity as approved by the Uganda Research Ethics Committee.] UGANDA ONLY

Name of Research Participant (please print) Date

Signature of Participant/Thumb print if participate is illiterate

Signature of Staff Obtaining Consent Date

**If participant is illiterate and a witness is available:**

I certify that _______________________ (participant’s name) has been read the consent on ____________

(today’s date) has had any questions answered and voluntarily agreed to take part in this study.

______________________ ___________________________ _____________

Witness (if participant is illiterate) Witness signature Date

YOU WILL BE GIVEN A COPY OF THIS CONSENT FORM TO KEEP.
